# Biological and Chemical Processes that Lead to Textile Malodour Development

**DOI:** 10.3390/microorganisms8111709

**Published:** 2020-10-31

**Authors:** Florence Van Herreweghen, Caroline Amberg, Rita Marques, Chris Callewaert

**Affiliations:** 1 Center for Microbial Ecology and Technology (CMET), Ghent University, Sint-Pietersnieuwstraat 33, 9000 Ghent, Belgium; florence.vanherreweghen@ugent.be; 2 Swissatest Testmaterialien AG, Mövenstrasse 12, 9015 St. Gallen, Switzerland; Caroline.amberg@swissatest.ch (C.A.); rita.marques@swissatest.ch (R.M.)

**Keywords:** malodour, textile odour, permastink, skin microbiome, volatilome

## Abstract

The development of malodour on clothing is a well-known problem with social, economic and ecological consequences. Many people still think malodour is the result of a lack of hygiene, which causes social stigma and embarrassment. Clothing is washed more frequently due to odour formation or even discarded when permastink develops. The malodour formation process is impacted by many variables and processes throughout the textile lifecycle. The contact with the skin with consequent transfer of microorganisms, volatiles and odour precursors leads to the formation of a distinctive textile microbiome and volatilome. The washing and drying processes further shape the textile microbiome and impact malodour formation. These processes are impacted by interindividual differences and fabric type as well. This review describes the current knowledge on the volatilome and microbiome of the skin, textile and washing machine, the multiple factors that determine malodour formation on textiles and points out what information is still missing.

## 1. Introduction

During their lifecycle, clothing items can become a source of malodour generation. This odour build-up has an effect on the daily life comfort, the life cycle of textile and the use of resources [1]. A survey by Kubota et al. [2] reported that 86% of respondents (580 women living in Tokyo) had encountered laundry malodour problems.

**Figure 1:**
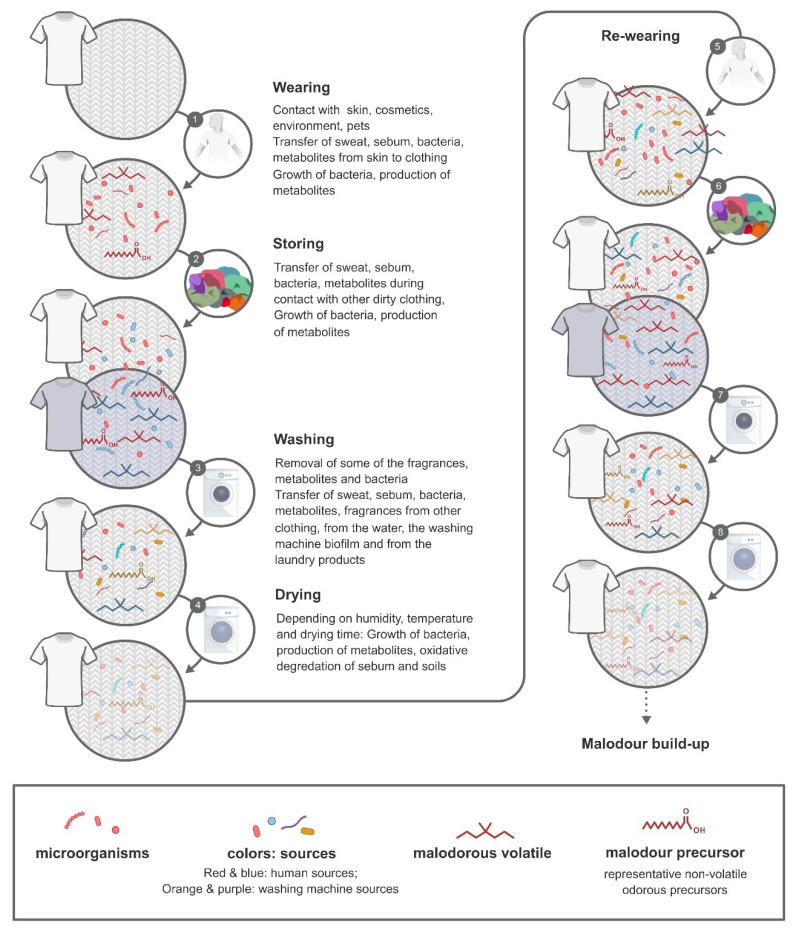
Schematic overview of different processes (wearing, storing, washing, drying, re-wearing) during two wear-wash-dry cycles affecting metabolites and microorganisms from different sources attaching to clothing. During the wearing (1), the clothing is in contact with the skin where a transfer of e.g., microorganisms and sebum occurs (indicated in red). When the clothing is stored before washing (2), a cross-over of these microorganisms and metabolites between clothing from another individual (indicated in blue) can take place. The washing process (3) reduces the amount of microorganisms and metabolites on the clothing, with an efficiency dependent on temperature, wash time and type of detergent. Furthermore, bacteria and metabolites present in the washing machine and in the wash water as well as fragrances and enzymes and other ingredients from the detergent/softener attach to the clothing. The effect of the drying process (4) is dependent on humidity, temperature and drying time, although relatively unstudied. In subsequent wear cycles (5) these processes are repeated and microorganisms and malodour volatiles accumulate to a situation of excessive malodour build-up or permastink.

These unpleasant odours can originate from many different sources and the development is influenced by a number of variables (Figure 1). During the wearing process, skin bacteria attach to the clothing and selective bacterial growth is possible. Sweat, sebum and bacterial metabolites are adsorbed on the clothing through contact with the skin and can serve as precursors for malodour. This leads to the formation of a distinctive textile microbiome and volatilome, which are impacted by the fabric type, the individual and the wearing process. The collection and storing of dirty laundry, before washing, allows for a transfer of bacteria and odorous compounds between different items of clothing, from different people.

Laundry, the washing of clothing, has been part of human domestic life for a long time and its goal is to remove dust, dirt and stains from the textile. Next to this more visible task, the washing process should also reduce the amount of bacteria and their metabolites present on fabric. This is not only important for hygienic reasons but also to diminish textile malodour formation. By washing at higher temperatures (>50 °C), for longer periods of time or by using oxidizing compounds (such as chlorine and activated oxygen bleach), the microbial reduction is typically sufficient to obtain adequate hygiene [3,4,5]. Whether this is also sufficient to obtain adequate odour removal remains unclear.

Due to energy conservation measures, the temperature of household washes were reduced in recent decades, whereas the wash duration increased [4]. An increased sensitivity of many modern textiles towards higher temperatures and chemical detergents likewise changed the washing habits. Washing processes at lower temperatures (~30 °C) with enzyme-based detergent free of oxidizing compounds are not reducing the microbial load on the fabric, but result in a microbial exchange between the microbiome in the washing machine, in the water and on the pieces of clothing [4,5,6,7]. Part of the odorants can be removed during washing, depending on fibre and individual, and can be replaced by fragrances from the laundry detergent and conditioner [8]. Reduced washing efficiency can potentially cause build-up of microorganisms and odour. This may have contributed to the increasing complaints about clothes that have a “permastink”, or about a dank smell of freshly washed clothes, of the washing machines and of poorly dried washed clothes [6,9]. Little research has been done focussing on the effect of the drying conditions on malodour formation, but factors like temperature, humidity and UV light will affect the evaporation of volatiles and the growth and activity of malodour-forming bacteria [2,6,10].

This article reviews the literature relating to odour formation on textile and the contributing factors. The aim of this review is to show the complexity of this multifaceted problem, to describe the variety of variables impacting this process and to point out what information is still missing.

## 2. The Human Skin

### 2.1. Skin Microbiome

One important contribution that shapes the textile microbiome is the skin microbiome, as clothes are in very close contact with the skin. In general, the skin is slightly acidic (pH = ±5.6), a bit cooler than the core body temperature (T = ±30 °C), relatively salty and dry. Distinct skin sites are determined by skin thickness, folds, density of hair follicles and glands, resulting in microenvironments with specific pH, moisture, sebum content, temperature and topography [11,12,13]. The microbial communities differ greatly in composition, diversity and abundance depending on these microenvironments. Moist microenvironments, such as axillae, perineum and toe webs, are occluded and higher in temperature and humidity. They have the highest bacterial counts, with up to 10^6^ organisms/cm², of which most species belong to the *Staphylococcus* or *Corynebacterium* genus or Betaproteobacteria class. Sebaceous microenvironments, on the upper body part, produces the lipid-rich sebum, which encourages the growth of lipophilic microorganisms such as *Cutibacterium* and *Malassezia*, as well as some *Staphylococcus* and *Corynebacterium* sp. Dry microenvironments are the most diverse regions with species from Betaproteobacteria class, *Corynebacterium* and *Cutibacterium* genus and Flavobacteriales order, and contain low bacterial counts (10³ organisms/cm²) [12,13,14,15].

These microenvironments not only determine community composition but also the temporal variability. Sites that are partly occluded and sebaceous, such as the auditory canal, the groin, and parts of the nose, are the most stable over time with respect to community membership and composition, whereas dry sites like the forearms, shins and hands show more variability. Temporal variability was shown to be modestly correlated with community diversity, as high diversity sites were likely to be less stable over time compared to low diversity sites [13,15,16,17].

Other factors influencing the microbiome are gender [18,19], geographical location [18,20], ethnicity [21], depth within the epidermis [12,19], antibiotic treatment [22], use of cosmetics [23,24,25], genetic disposition [26], age [18,25] and health status [27]. Despite the high interindividual and temporal variability, the skin microenvironment is the primary factor describing the variation in the community structure [14,17,18]. One good example is the armpit, where a high concentration of eccrine, apocrine and sebaceous sweat glands, hair and the occluded nature leads to the growth of high abundance of bacteria, which may be associated with malodour formation [28].

### 2.2. Body Odour

Body odour, or malodour generation on different body sites, is caused by the microbial degradation of skin secretions. Members of the skin microbiota are capable of transforming the odourless secretions from eccrine, apocrine and sebaceous glands into volatile odorous molecules [29,30,31]. The warm, moist and nutrient rich environment of the axillae ensures the continuous colonization by an abundant and diverse microbial population, making the axillary microenvironment a hotspot for odour formation.

The odour profile is dependent on the microbial community composition and the quality and quantity of the secretions and is thus impacted by age, gender, cosmetics use, diet, climate, stress, hygiene etc. [28,31,32,33,34]. Studies have shown that corynebacteria abundance is correlated with body malodour and that *Corynebacterium tuberculostearicum*, *Staphylococcus hominis* and *Anaerococcus* sp. are important actors in odour formation [28].

The odour precursors mainly come from the apocrine secretions that consist of long-chain fatty acids, fatty acids bound to amino acids, sulphur containing amino acids, vitamins and steroids. The eccrine glands are responsible for thermoregulatory sweat secretion and release a more watery electrolyte solution containing Na^+^, K^+^, Ca^2+^, Mg^2+^, Cl^-^, HCO3^-^, lactate, urea and ammonium [30,35,36].

Based on the current literature we can distinguish different microbial transformations resulting in odorous volatiles: steroid-, sulphur- and volatile fatty acids (VFA)-based malodours. Sulphurous compounds have a low olfactory threshold and contribute greatly to the axillary odour, giving it its typical onion-like and musky scent. Bacterial dipeptidases and C-S-lyases can cleave the carbon–sulphur bonds in the cysteine-based conjugates precursor (e.g., S-hydroxyalkyl-L-cystein(glycine)) resulting in the release of mercaptoalcohols, such as 3-methyl-3-mercaptohexan-1-ol [28,36,37,38]. Both corynebacteria and staphylococci can carry out these transformations, and studies have identified *Staphylococcus hominis*, *Staphylococcus haemolyticus* and *Staphylococcus lugdunensis* to be particularly efficient [39,40].

Other microbial transformations lead to the production of short-, medium-, and branched-chain volatile fatty acids, which contribute to the odour profile by evaporation or by promoting microbial growth. Lactic acid and glycerol, released from triacylglycerides, can be converted by *Staphylococcus* and *Cutibacterium* species to acetic acid and propionic acid [30,41]. Biotransformation of leucine, or other branched aliphatic amino acids, by *Staphylococcus* species results in isovaleric acid, an important contributor to the acidic note of axillary malodour. These amino acids are present in eccrine sweat, but may also come from bacterial degradation of proteins in apocrine secretions or keratinising epidermis. Another route to short and medium chain VFAs is through N-acylglutamine aminoacylases activity by *Corynebacterium* species. This enzyme cleaves compounds such as 3-methyl-2-hexenoic acid (3M2H) and 3-hydroxy-3-methylhexanoic acids from their glutamine conjugate [29,30,42]. *Corynebacterium* species can also contribute to VFA-based malodour through partial degradation of methyl-branched long-chain fatty acids present in sebum. However, the share of this β-oxidation pathway in the VFA production may be less significant than previously thought, as the amount of precursor is limited as is the number of bacterial species able to carry out this β-oxidation [29,30]. Same goes for the biotransformation of steroids, which was previously suggested to be heavily implicated in axillary malodour, but more recent studies found very few axillary microorganisms capable of this transformation. Furthermore, 50% of the population is anosmic for these compounds [43].

## 3. Clothing

### 3.1. Textile Volatilome

Clothing contributes to odour intensity since secretions, skin debris, sebum, odorous volatiles and microorganisms are transferred from the body to the garment (Figure 1). It was stated by Shelley et al. [31] that odour intensity is potentially more intense in the fabric substrate than in the adjacent axillae. Dravnieks et al. [44] has discriminated between a primary odour, which originates in the axilla itself, and the secondary odour developing on the garments due to abiotic or biotic processes. UV-light, temperature and chemical reactions (e.g., due to detergent compounds, fabric softeners, enzymes) can transform substrates into odorous volatiles [43]. Microorganisms attaching on the fibres are able to use dirt or sebum compounds as substrate and generate volatiles as by-products [6,45,46].

Human sebum is the major constituent of the organic soil load on textiles [47]. Some sebum components are difficult to remove by a washing process especially from hydrophobic fabrics such as polyester [48]. As a result, fatty components remain on the fabric and form a potential substrate for microbial growth and odour formation.

In general, unwashed textiles have a volatilome close to the one generated in the axilla with the predominance of short chain fatty acids, as isovaleric acid, and branched-chain fatty acids like 3-hydroxy-3-methylhexanoic acid or 3-methyl-2-hexenoic acid [46]. However, given by the distinct surface properties and functional groups, different fibre types have a selective uptake and retention of volatile substances. The adsorption of odorous volatiles is impacted by the polarity of odorant and fabric, as well as by the hydrophobicity of the fibre. Cotton is polar and sorbs aldehydes in high quantities, while polyester, due to its lower polarity, adsorbs less moisture [49,50]. On the other hand, the hydrophobic nature of polyester results in a strong adherence of fatty acids and aromatic compounds [50].

The numerous reactive sites of natural fibres (hydroxyl-groups in cellulose, amino acid side chains in wool) may also play a role in textile odour formation by offering adsorption sites for volatiles. It is conceivable that the odorous compounds are “trapped” in natural fibres and easier released from polyester [51,52]. This is in line with the study by Hammer et al. [53] where retention of isovaleric acid was lowest on polyester in comparison to cotton and wool. Recent work by Abdul-Bari et al. [54] showed that cotton sorbed greater amounts of octanoic acid compared to polyester, whereas polyester sorbed more nonenal than cotton. Despite the high absorption and affinity of octanoic acid to cotton, laundering removed more octanoic acid from cotton than from polyester. Moreover, nonenal was more effectively removed from cotton. Odour quality and intensity were judged consistently different across different fibre types. Polyester and polyester blends exhibit, in general, higher malodour intensities in comparison to cotton and wool [45,55,56]. The hedonic values of polyester were qualified as more negative (= more unpleasant) than cotton, with higher values for the odour characteristics “sweaty”, “sour”, “musty” and “ammonia” [23].

Mass and structure of fabrics have also some impact on odour formation. As expected, fabrics with a lower mass per m^2^ and a plain structure, exhibit lower odour intensities [55,57]. A washing process alters the volatilome of textiles and adds some compounds to the textile, such as detergent or softener ingredients, soil, volatiles and microorganisms from the washing machine or from other textiles. After washing, aldehydes are the predominant volatile compounds, whereas the low molecular weight organic acids were only found in smaller numbers on the washed laundry [6,46]. Aldehydes contributed most importantly to the overall odour of laundry extracts. A compilation of volatile compounds found on unwashed / washed textiles is presented in Table 1.

### 3.2. Textile Microbiome

The contribution of microorganisms on the generation of the secondary odour on textiles is still unclear. In the underarm region, bacteria are transferred in high numbers to the textile due to the close permanent contact, transferred via sweat. Mixed cultures of bacteria from the axilla were able to form biofilm on textiles [69]. However, axillary and textile microbiomes differ in their compositions. Even though the same main phyla Firmicutes, Actinobacteria and Proteobacteria were found on worn textiles and in the axilla, some synthetic textiles seem to promote attachment and growth of particular taxa that are underrepresented in the axillae [70]. While the axillary skin microbiome is dominated by *Staphylococcus* sp. and *Corynebacterium* sp., textile microbiome consists mainly of *Staphylococcus* sp., *Micrococcus* sp., and to a lower extent of *Bacillus* sp., Enterobacteriaceae, and *Acinetobacter* sp. Corynebacteria, key microorganisms for sweat odour formation in the axilla, could not be isolated from worn textiles [23,55,70]. Similarly as the axillary microbiome, the textile microbiome is rather individual in its composition. It seems that different microorganisms and microbial pathways are relevant for secondary odour generation. Alternatively, textile odour formation can be influenced by, for example, the physical and chemical attributes of the textile.

Different fibre types have different surface properties and functional groups which impact not only adsorption and retention of volatile compounds, but also bacterial attachment and growth. Several studies reveal a selective attachment of microorganisms on different fibre types. *Staphylococcus* sp. was enriched on more or less all fibre types [23,70,71], with *Staphylococcus hominis* having a high affinity to cotton [23]. *Micrococcus luteus* was accumulated on polyester, polyester blends and wool [23,70]. Wool promoted growth of many bacteria, such as *Enhydrobacter* sp., *Cutibacterium* sp., *Staphylococcus epidermidis* and *Micrococcus* sp. [23]. Cellulose-based fibres such as viscose or Tencel™ showed a low microbial growth potential for most axillary bacteria except for some *Staphylococcus* sp. [23,70]. *Corynebacterium jeikeium*, was not able to enrich on cotton, acrylic, wool, viscose, nylon, fleece and polyester, which is congruent with the findings that only low numbers of *Corynebacterium* sp. could be isolated from worn textiles [23,70].

## 4. Washing Machine

### 4.1. Impact of Washing Process on Volatiles and Microorganisms

The very essence of the laundry process is to obtain clean and fresh clothes, free from soil, dirt, pathogens and malodour. The washing process removes microorganisms and odorous volatiles, such as short chain fatty acids, from the fabrics. Detergent compounds and fragrances attach to the fibre during the washing cycle and contribute to textile odour intensity and profiles. When textiles undergo several wash and wear cycles, the resulting malodour has been influenced by several parameters like initial soil and microbe level of the garments, the storage environment, the washing and drying processes and the cleanliness of the washing machine (Figure 1).

Overall, the odour intensity is decreased by the laundering process [8]. Due to environmental reasons however, the washing temperature and water consumption have substantially declined in the last decades. Frequent use of low temperature cycles, with low water consumption and activated oxygen bleach (AOB)-free colour detergents, were shown to be inefficient in removing microorganisms, sebum and odorous compounds, and may contribute to the build-up of malodour on textiles and washing machines [6]. Furthermore, liquid detergents without activated oxygen bleach (AOB) have gained more popularity due to their convenience. Washing performance (soil removal) could at least partly be compensated by longer washing programmes and the use of enzymes. Unfortunately, it is not that easy for the removal of microorganisms. Honisch et al. [3] have systematically investigated the impact of temperature, time and detergent on microbial reduction of different bacterial and fungal strains on textile pieces in a household washing machine. Since microbial reduction is a synergistic result of time, chemistry, temperature and water consumption, it is not possible to assess the distinct effects of the single factors. In general, temperatures of 50 °C and above lead to a sufficient bacterial removal in household washing processes, independent from the used detergent and programme [5,72,73,74]. The use of AOB-containing detergents can compensate to a certain extent for lower temperatures. Fungal strains on the other hand, are more difficult to inactivate and some are rather resistant toward AOB-containing detergents [75].

Odour-removal efficiency of a washing process is fibre-dependent. Washed polyester still exhibits higher odour intensities than washed cotton or wool, without having differences in bacterial counts (total microbial counts, aerobic *Corynebacterium* sp.). It was shown that fatty acids, aldehydes and aromatic compounds were much easier removed from cotton than from polyester [45,54]. The higher moisture regain and hydrophilicity of cotton allows the laundry liquid to enter the fibre structure and breaks the interactions between cotton fibre and odorants. This could be a further factor for the cumulative odour build-up on polyester over multiple wash and wear cycles and could refer to the so-called “old-sweat odour”, or “permastink”, respectively, the ‘sportswear problem’ [1,23].

In two Japanese studies, 4-methyl-3-hexenoic acid (4M3H) was identified as a frequently detected odour component of washed and smelly laundry. The most prominent species found on those laundry items was *Moraxella osloensis* and its ability to produce 4-methyl-3-hexenoic acid was proven [2,76]. Furthermore in a recent study in German households, *M. osloensis* was identified as an abundant colonizer of the washing machines [77]. The precursors for the conversion into 4M3H are not known and are a subject for further investigation.

Laundry habits do differ across the world. Many people still do hand washes with soap. Most European washing machines are horizontal axis machines and usually wash at a temperature of 30 °C to 60 °C, with an average temperature in the European Union of 42.7 °C [78]. In South and North America and a lot of Asian countries, vertical axis washing machines (top loaders) are common. They run either with cold (~10–20 °C) or hot water, while the average washing durations are shorter in the US (35 to 85 min for top-loaders) as compared to Europe (70 to 120 min for front-loaders) [79,80]. In Japan, short and cold wash cycles are used frequently and the bath water might be reused for laundry washing [2,43]. In some countries (like Belgium [7]), rainwater is collected from the roof and used as influent water.

### 4.2. The Washing Machine Volatilome and Microbiome

Washing machines provide perfect living conditions for microorganisms due to the humidity and the nutrients present. Nutrients and microorganisms are introduced through water, dirty laundry, washing machine biofilms and human handlers. The microorganisms are able to survive and thrive through biofilm formation and stagnant water. Microorganisms making up these biofilms include bacteria of the genera *Acinetobacter*, *Bacillus*, *Brevundimonas*, *Micrococcus*, *Staphylococcus* and *Pseudomonas* and fungi from the *Candida*, *Fusarium*, *Aspergillus* and *Trichosporum* genera [81,82,83]. Biofilms are typically formed in hardly accessible, wetted parts that are difficult to clean, such as the detergent drawer, the rubber door seal, the filter [6,82], inside plastic and synthetic parts of the washing machine and inside stagnant water of the washing machine. Gattlen et al. [82] sampled eleven washing machines from four countries and three continents and found that while the bacterial load (plate counting) was in the same range, the bacterial composition differed greatly between washing machines. Different laundry habits across the world also impact the biofilm composition. Other factors impacting the washing machine microbiome are influent water, clothing, soils, bacterial exchange, climate, as well as number and age of household members.

Mild washing conditions may result in an enrichment of opportunistic and/or antibiotic-resistant pathogens in washing machine biofilms as well as in increasing malodour formation [3,82,83,84,85,86]. Leaving washed laundry in the washing machine overnight, leads to an enrichment of the biofilm-related *Pseudomonas* sp. on washed clothes [7].

Stagnant water in tubing or inside the washing machine is a source for bacterial and fungal growth, and consequently odour formation [67]. A wide range of volatiles, such as 1,3-dioxanes, 1,3-dioxolanes, alkyl sulphides, geosmin, 2-methyl isoborneol, trans-2-cis-6-nonadienal, 2,3,6- trichloroanisole, 2-isopropyl-3-methoxypyrazine, thiophene, dimethyl sulphide, dimethyl disulphide and carbon disulphide, have been identified as off-odours in influent and effluent water streams [67].

Stapleton and Dean [66] investigated the possible impact of washing machine malodour on overall textile malodour and propose that washing machine malodour compounds contribute to a high extent to the overall textile malodour. Volatile compounds as well as bacteria are probably transferred from contaminated washing machines to laundry fabrics. This is, for instance, sometimes noticed by a typical musty odour when clothes are left in humid conditions in the washing machine after laundering.

To prevent biofilm build-up and associated malodours, most washing machine producers advise a frequent maintenance wash, with high temperatures and a bleaching agent. Some newer machines also include special treatments that are advertised to improve removal of microorganisms and malodour from both clothing and washing machines: for example, by using silver ions (Samsung), steam (Whirlpool) and even ultrasonic waves (Sanyo). However, studies critically evaluating these approaches have not yet been performed and actual efficacy remains to be seen.

## 5. Factors that Determine Malodour Formation

One important factor is the microbiome. Selective enrichment of microorganisms on clothing textiles and in washing machines are important reasons for textile malodour formation. *Micrococcus* sp. [29,70] and *Moraxella* sp. [76] have already been identified and linked. Other microbiota will likely play an important role, but further research is needed to identify them. Unlike the composition of the microbial community, the microbial load does not seem to be a determining factor [45,55,87]. As a result, antimicrobial coatings on textile have been investigated as a way to prevent odour formation. However, it was shown in several studies that antimicrobial coatings do not necessarily lead to an odour decrease [60,88], and they could affect the skin microbiome (unpublished data).

Another contributing factor is metabolite build-up in clothes and washing machines. The metabolite load in clothing textiles can increase over time, as microbial and environmental degradation of hydrophobic apocrine secretions, sebaceous secretions, skin desquamations and other human sources, can adhere and bind to clothes which leads to a build-up of metabolite precursors [89]. It is known that dry and freshly washed permastink clothes emit a direct malodour when ironed, which can be explained by the release of covalently bound hydrophobic molecules that are released with the heat. Additionally, both type and quantity of metabolites can differ greatly between individuals. A study by Martin et al. [26] showed that an alteration in the human ABCC11 gene, which is prominent among Asian people, impacted apocrine sweat secretions and thus metabolite build-up. Other interindividual dissimilarities, among which gender, body mass index, hygiene habits, ethnicity, nutritional and intercultural differences likely play a role in metabolite build-up in clothes. The identity and origin of these metabolites are nonetheless still the subject of future studies. Laundry detergent ingredients should focus on removal of those hard-to-remove metabolites. Incorporation of activated carbon, zeolites, and cyclodextrins [90] have been suggested.

The fibre type is an important aspect in the malodour build-up process, as different fibres will react differently to the wear, wash and dry processes during the textile life cycle. Synthetic fibres, such as polyester, nylon and polyamide, are apolar, have hydrophobic functional groups and can adhere lipids and fatty acid molecules well [50]. Malodour precursors are likely more easily removed from natural fibres in the laundry process. The large interstitial surface and absorbing capacity of natural fibres can additionally contribute to a differential odour release during wear. A promising solution might be the modification of surface properties of synthetic fibres such as the application of hydrophilic coatings [53]. However, optimal solutions should be found for each textile type and should also cover fibre blends, re-used and up-cycled fibres.

The washing process has a big influence in odour removal, which is strongly influenced by time, temperature, detergent type/ingredients, wash load, fabric softeners, and frequency of washing. Due to the growing use of non-bleach containing liquid detergents and the ongoing trend towards cold-wash conditions, hygiene performances of washing processes have decreased. Only in hospital settings, it is still preferred to use bleach-based detergents and concomitant high temperatures [91,92,93,94,95]. Additionally, it was found that lipase-containing detergent led to a higher perceived odour intensity and qualitative differences in aldehyde compounds, as compared to the same detergent without lipase [6]. Likewise, the use of fabric softeners seems to increase the malodour problem on polyester and synthetic blends, whereas there is no odour impact of fabric softener on cotton fabrics [96]. An optimal program and detergent combination and a monthly washing machine cleaning measure can lead to an improved laundry efficacy.

The drying conditions additionally play an important role in the load of microorganisms and metabolites present on the dried textiles. Humidity after washing and during drying is an important contributing factor. Fast drying and tumble drying were found to be effective in decreasing the microbial load, while slow drying resulted in a higher microbial load on textiles [6,97,98]. Laundered clothes that are left in humid conditions are known to form immanent malodours. Moreover, drying outdoors and in sunlight seems to have a positive impact in reducing microbial load and malodour [98]. The UVB (~310 nm) in natural sunlight is known to reduce the fungal load [99] and likely other microbiota in clothes. UVB light generates oxidative free radicals that can actively attack macromolecules such as lipids and sweat secretions [100]. Under these circumstances, covalently bound metabolites on textiles can be released from their permanent position. As a practical example, commercial washing machines are available on the market that incorporate UV light into their drum. A steady and fast drying is advised, preferably outdoors and in sunlight, to prevent malodour formation.

## 6. Conclusions

In this review we combined the current knowledge on different aspects and impacting factors on textile malodour formation and build-up.

There is a clear lack of understanding of how the microbiome impacts malodour formation on clothes and in washing machines. Not only a qualitative description of bacterial factors, by means of sequencing technology, but also the activity of the bacteria by means of metatranscriptomics, and the involved genes and the pathways leading to malodour, by means of metagenome prediction from short read technology and/or metagenomic analysis from whole genome sequencing. In addition, it is important to learn more about the involvement and influence of fungi, archaea and micro-eukaryotes in clothing and malodour development. Studies have mainly identified malodorous volatiles using GC/MS. It would be interesting to perform untargeted metabolomics (with LC/MS), to identify the larger molecules present on clothes and in washing machines, in order to identify the precursors and the possible biological pathways.

The current knowledge mainly comes from smaller studies and experiments, focussing on one or few aspects of the malodour formation process. While these results offer useful and important findings, the conclusions are limited due to their limited scope. There is a need for larger scale studies focusing both the composition and quantity of the microbiome and volatilome throughout the entire wear–wash–dry cycle of clothing. Taking into account the interindividual differences as well as different fibre types, different wearing, washing and drying conditions, can offer more insights into the different factors interacting and impacting the malodour formation process. These insights are much needed to open up opportunities for the development of new techniques and approaches to combat clothing and laundry malodour formation.

## Figures and Tables

**Table 1:** Odorous compounds identified on textiles unwashed after wear and/or after a household laundering process. FD = flavour dilution. Some studies show data on microorganisms and / or volatiles of clothes after wearing and before washing (= after wearing), while other studies present data on worn clothes after washing (= after washing). “Involved microorganisms” refers to microorganisms that have been linked with the production of the respective substances in the literature.

Volatile Group	Odorous Volatiles	Involved Microorganisms	Analysis before and/or after Washing, FD-value	Literature
Fatty acids	Ethanoic acid (acetic acid)	*Propionibacterium sp.* *Staphylococcus sp.*	after wearing	[41,58]
	Propanoic acid	*Propionibacterium sp.* *Staphylococcus sp.*	after wearing	[41,58]
	2-methylpropanoic acid (isobutyric acid)	*Bacillus subtilis*	after wearing	[59]
	Butanoic acid		after wearing	[8]
	2-methylbutanoic acid	*Bacillus subtilis*	after wearing	[59]
	3-methylbutanoic acid (isovaleric acid)	*Bacillus subtilis* *Staphylococcus epidermidis*	after wearing and after washing	[6,38,43,59,60,61,62]
	3-methyl-2-hexenoic acid	*Corynebacterium sp.* *Micrococcus sp.*	after wearing	[42,58,60,63,64]
	4-methyl-3-hexenoic acid (4M3H)	*Moraxella osloensis*	after washing	[2,43]
	5-methyl-4-hexenoic acid		after washing	[43]
	3-methyl-3-hydroxy-hexanoic acid	*Corynebacterium bovis, Corynebacterium jeijeikum, Corynebacterium striatum*	after wearing	[42,60,63,64]
	6-heptenoic acid		after washing	[43]
	4-methyloctanoic acid		after washing	[6]
	4-ethyloctanoic acid		after washing; FD > 256	[6]
Steroid compounds	5-α-androstenol		after wearing	[65]
	5-α-androstenone		after wearing	[66]
	5-α-androst-2-en-17-one	*Staphylococcus sp. Corynebacterium sp.*	after wearing and after washing	[6,38,60,61,62,67]
	5-α-androst-16-ene-3-one		after wearing	[58]
Sulfur compounds	3-methyl-3-sulfanyl-hexan-1-ol (3M3SH)	*Staphylococcus haemolyticus; Staphylococcus hominis*	after wearing	[38,39,42,60,63,64]
	Dimethyl disulphides		after washing	[8,68]
	Dimethyl trisulphides		after washing	[8,68]
	Benzyl mercaptan	*Corynebacterium* sp.	after wearing	[39]
Ketones	1-hexen-3-one		after washing	[6]
	2-heptanone		after wearing	[8]
	2-octanone		after wearing	[8]
	1-octen-3-one		after wearing and after washing	[6,67]
	2-nonanone		after wearing	[8]
	Medium-chain ketones (undetermined)			[10]
Esters	Ethyl-2-methylpropanoate		after washing; FD > 256	[6]
	Ethyl butanoate		after wearing and after washing; FD > 256	[6,67]
	Methyl-3-methyl-hexanoate		after wearing	[58]
	Methyl laurate		after wearing	[58]
	Methyl myristate		after wearing	[58]
	2-Aminoacetophenone		after wearing; FD > 256	[6]
	Diethyl phthalate		after wearing	[58]
Aldehydes	Methional		after washing	[6]
	Hexanal		after washing	[46]
	(Z)-4-heptenal		after washing; FD > 256	[6]
	Octanal		after washing; FD > 256	[6]
			after washing	[46]
	(E)-2-octenal		after washing	[6]
	Cis/trans-2-nonenal		after wearing and after washing; FD > 256	[6,46]
	(E,Z)-2,4-nonadienal		after washing; FD > 256	[6]
	(E,Z)-2,6-nonadienal		after washing; FD > 256	[6]
	Decanal		after washing	[43]
	(E,E)-2,4-decadienal		after washing	[6]
	(E)-4,5-epoxy-E-2-decenal		after wearing; FD > 256	[6]
	4-methoxybenzaldehyde		after washing; FD > 256	[6,68]
	Medium-chain aldehydes (not determined)			[10]
Alcohols	Oct-1-en-3-ol		after washing	[67]
	2-Nonanol		after wearing	[58]
	1-Decanol		after washing	[68]
	1-Dodecanol		after washing; FD > 256	[68]
	2-Phenylethanol		after wearing and after washing; FD > 256	[67,68]
	2-Methoxyphenol (guaiacol)		after wearing and after washing	[6,67]
Others	Naphthalene		after wearing	[67]
